# Racial/ethnic differences in social determinants of health and health outcomes among adolescents and youth ages 10–24 years old: a scoping review

**DOI:** 10.1186/s12889-023-15274-x

**Published:** 2023-03-01

**Authors:** Patricia Monroe, Jennifer A. Campbell, Melissa Harris, Leonard E. Egede

**Affiliations:** 1 Center for Advancing Population Science, Medical College of Wisconsin, 8701 Watertown Plank Rd, Milwaukee, WI 53226 USA; 2 Division of General Internal Medicine, Department of Medicine, Medical College of Wisconsin, Milwaukee, WI USA

**Keywords:** Scoping review, Social determinants of health, Adolescence, Adolescence, Racial/ethnic differences

## Abstract

**Introduction:**

With the recent emergence of the Healthy People 2030 goals there is a need to understand the role of SDOH on health inequalities from an upstream perspective. This review summarizes the recent body of evidence on the impact of SDOH across adolescence and youth health outcomes by race/ethnicity using the Health People 2030 Framework.

**Methods:**

A systematic, reproducible search was performed using PubMed, Academic Search Premier, PsychInfo, and ERIC. A total of 2078 articles were screened for inclusion. A total of 263 articles met inclusion criteria, resulting in 29 articles included for final synthesis.

**Results:**

Across the 29 articles, 11 were cross-sectional, 16 were cohort, and 2 were experimental. Across SDOH categories (economic stability, education access and quality, health care access and quality, neighborhood and built environment, and social and community context), 1 study examined self-efficacy, 6 educational attainment, 10 behavior, 5 smoking, 11 alcohol use, 10 substance use, and 1 quality of life. The majority of outcomes represented in this search included health behaviors such as health risk behavior, smoking, alcohol use, and substance use. Across the 29 articles identified, significant differences existed across outcomes by race/ethnicity across SDOH factors, however magnitude of differences varied by SDOH category.

**Discussion:**

SDOH differentially affect adolescents and youth across race/ethnicity. The lived adverse experiences, along with structural racism, increase the likelihood of adolescents and youth engaging in risky health behaviors and negatively influencing health outcomes during adolescence and youth. Research, public health initiatives, and policies integrating SDOH into interventions at early stage of life are needed to effectively reduce social and health inequalities at a population level.

## Introduction

The impact of social determinants of health (SDOH) on the overall well-being of individuals and population health has been well established [1–4]. The World Health Organization describes SDOH as multiple factors impacting the health of individuals, including “structural determinants and conditions of daily living,” unequally around the world [5], which over the life course, contribute to the development of chronic illnesses, often among those most vulnerable [5–7]. The Healthy People 2020 Framework for SDOH established a foundation for examining the role of SDOH across health and well-being, fostering a substantial gain in knowledge regarding the contribution of SDOH on health and well-being of adults, including racial/ethnic disparities in SDOH [2, 4, 8–10]. With the recent emergence of the Healthy People 2030 goals to “create social, physical, and economic environments that promote attaining the full potential for health and well-being for all” there is a need to understand the role of SDOH on health inequalities from an upstream perspective, specifically the presence of SDOH in early life [11], that may contribute to adult morbidity.

Adolescence as a phase of development begins at age 10 and transitions to youth at age 19 through age 24, according to the WHO [12]. This period of development presents a unique time of transition, with increased awareness of social processes and vital brain development [12–14]. This transition is marked by development of new behaviors, influenced by societal contexts, resulting in positive or adverse health outcomes and ultimately, inequalities as adolescents age and transition into adulthood [12, 14, 15]. Evidence shows that SDOH and social risk factors such as access to care, health insurance, food security, access to transportation, neighborhood deprivation, and economic disadvantage have been found to negatively impact outcomes for racial/ethnic minority adolescents [16–22], lending to racial/ethnic health disparities across health indices. For example, higher rates of obesity and behavior problems, poorer cardiovascular and oral health, and lower rates of health-related quality of life [16–19, 21, 22].

While the existing body of evidence provides identification of key SDOH factors that lend to health disparities across adolescent and youth outcomes, systematic evaluation of the existing evidence for how SDOH impact the health, well-being, and the development of adolescents and youth across racial/ethnic groups has been limited [15, 23]. To address SDOH factors that lend to health inequalities for adolescence and youth, an in depth understanding of how SDOH contribute to health outcomes in adolescence and youth across racial/ethnic groups must first be established. This scoping review therefore aims to evaluate and synthesize the existing evidence for the role of SDOH on adolescent and youth health outcomes within the United States, and to summarize similarities and differences found across race/ethnicity. Specifically, using the Healthy People 2030 Framework as a guiding framework, the literature was searched matching terms to the SDOH domains of economic stability, education access and quality, health care access and quality, neighborhood and built environment, and social and community context, to evaluate and summarize existing evidence for the role of SDOH on adolescent and youth health and well-being across a broad spectrum of health indicators.

## Methods

### Information sources, eligibility criteria, and search

PRISMA Guidelines were used for identifying, screening, and study selection for final synthesis. No protocol was prepared for this review. Articles were chosen based on eligibility criteria listed below, established a priori by the authors. A reproducible search strategy was used to identify articles investigating the impact of SDOH on the health outcomes of adolescents and youth 10–24 years of age, based on the WHO definition [12]. Four different databases were utilized to ensure the inclusion of a robust set of articles. Articles published between 2014 and 2021 were searched using PubMed, Academic Search Premier, PsychInfo, and ERIC. This date range was chosen a priori to maximize applicability and relevance of evidence. Medical Subject Heading (MeSH) terms representing SDOH based on the Healthy People 2030 Framework were used, see Table 1, along with additional inclusion criteria (listed below, and Table 2).

**Table 1:** Search terms

MeSH Terms – SDOH	MeSH Terms – Outcomes	MeSH Terms – Characteristics
Social determinants of health Health social determinant Health social determinants Socioeconomic factor Socioeconomic status Socioeconomic gradient Socioeconomic position Low income Poverty Trauma Psychological trauma Stress Social support Social disparity Social environment Social exclusion Social factor Social gradient Social position Social cohesion	Self-efficacy Educational achievement Psychological resilience Behavior, health risk Smoking behaviors Alcohol drinking Substance abuse detection Health related Quality of life	Race factors Minority health Health status disparities Adolescent^a^ Young adult^a^

^a^searched as Boolean/phrase

**Table 2:** Inclusion, exclusion criteria

Inclusion	Exclusion
• At least one or more outcomes must be included as an outcome evaluated in study: ◦ Self-efficacy ◦ Educational attainment ◦ Psychological resilience ◦ Behavior, health risk ◦ Smoking behavior ◦ Alcohol use ◦ Substance use ◦ Quality of life • Racial/ethnic differences in outcomes must be presented • Type of study: ◦ Cross-sectional ◦ Cohort ◦ Clinical trial ◦ Quasi-experimental ◦ Pre-post • English language	• Disease specific focus • Population age: ◦ Younger than 10 years ◦ Older than 25 years • Type of study: ◦ Systematic reviews ◦ Meta-analysis ◦ Scoping review • Protocol, design, or rationale papers

Eligible articles were included based on the following inclusion criteria: 1) published in English, 2) based in the United States, 3) study design: cross-sectional, cohort, clinical trial, quasi-experimental, or pre-post study design, 4) outcomes demonstrated across at least two racial/ethnic groups showing outcomes were examined by race/ethnicity. Additionally, one or more of the following outcomes had to be included: 1) self-efficacy, 2) educational attainment, 3) psychological resilience, 4) health risk behavior, 5) smoking behavior, 6) alcohol use, 7) substance use, 8) quality of life. Health risk behavior, including high risk sexual behavior, delinquent behavior, and health promoting behavior including physical activity and dietary intake. Outcomes were chosen based on the evidence of health behaviors during adolescence and youth impacting morbidity and mortality in adulthood, as well as the potential impact of moderators, such as self-efficacy, educational attainment, and psychological resilience, throughout the life course on health outcomes [24–26].

### Study selection and data collection

**Fig. 1:**
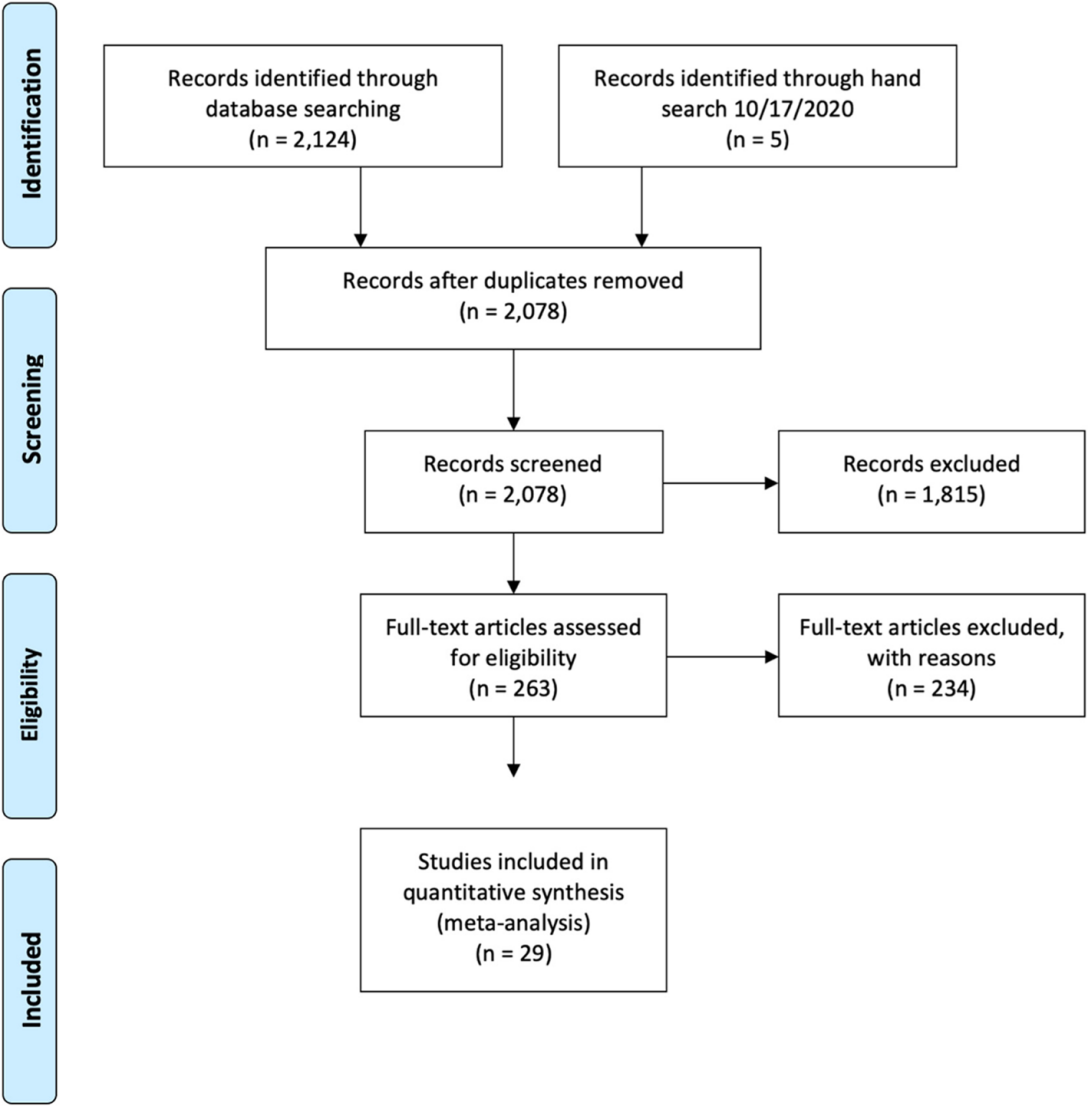
PRISMA Flow Diagram

Study selection was based on an initial title and abstract review by PM and MH. Studies were evaluated for inclusion using a checklist that included eligibility criteria. Studies not meeting eligibility criteria were excluded. After the title and abstract review, full text articles that met initial inclusion criteria were included for full text synthesis. Initial and full text review of studies were done separately with oversight provided by JAC and LEE. The checklist ensured consistent decision-making processes were followed for each paper reviewed. After full text synthesis by PM, MH and JAC, articles not meeting inclusion criteria were excluded with reasons. Please see Fig. 1 for PRISMA guidelines with details of studies excluded and retained at each phase. The articles included for data extraction are shown in Table 3. Data extraction included the study design, SDOH category, and outcomes assessed. Data quality was assessed using the JBI critical appraisal checklist [27]. JBI provides checklists by study design. This review used the appropriate checklist for the appropriate design in each paper. Final article decisions were made by PM, JAC, and LEE based on the checklists and included articles meeting all criteria to ensure quality across articles summarized in this review.

**Table 3:** Study design, race/ethnicity, and outcomes

Author/Year	Study Design			Race/Ethnicity							Outcomes							
	Cross-sectional	Cohort	Experimental	African American	Hispanic	Non-Hispanic White	Asian/Pacific Islander	American Indian	Other race/ethnicity	Multiracial	Self-Efficacy	Educational Attainment	Psychological Resilience	Health Risk Behavior	Smoking	Alcohol	Substance Use	QOL
Abram et al. 2017 [28]		X		X	X	X						X					X	
Assini-Meytin et al. 2019 [29]		X		X	X	X						X				X	X	
Baiden et al. 2019 [30]	X			X	X	X	X		X					X		X		
Bares et al. 2019 [31]	X			X	X	X											X	
Benner & Wang, 2014 [32]		X		X	X	X	X		X			X						
Benner & Wang, 2015 [33]		X		X	X	X	X									X	X	
Bohnert et al. 2017 [34]			X	X	X									X				
Brooks-Russell et al. 2019 [35]	X			X	X	X			X						X	X	X	
Cage et al. 2018 [36]		X		X	X	X			X			X						
Camenga et al. 2018 [37]		X				X			X						X			
Chambers et al. 2018 [38]		X		X		X								X			X	
Champion et al. 2016 [39]			X	X	X									X	X	X	X	
Chong et al. 2019 [40]	X					X			X		X							
Clark et al. 2015 [41]		X		X		X										X		
Combs et al. 2018 [42]	X			X	X	X		X		X		X						
Docherty et al. 2018 [43]		X		X		X								X				
Gerard & Booth 2015 [44]		X		X	X	X				X				X				
Hai 2019 [45]		X				X			X							X		
Hatchel & Marx 2018 [46]	X					X			X								X	
Hussong et al. 2018 [47]		X		X		X								X	X	X		
King 2017 [48]		X		X	X	X	X	X						X				
Komro et al. 2016 [49]	X					X		X								X		
Kucheva 2018 [50]		X		X		X						X						
Leventhal et al. 2018 [51]		X		X	X	X	X		X	X					X	X	X	
Lindberg et al. 2019 [52]	X			X	X	X			X					X				
Respress et al. 2018 [53]	X			X		X			X					X				
Santa Maria et al. 2018 [54]	X			X	X	X			X	X						X	X	
Wade & Peralta 2017 [55]	X			X		X										X		
Wallander et al. 2019 [22]		X		X	X	X												X

^a^*QOL* Quality of Life

## Results

### Study selection

Figure 1 shows the PRISMA diagram with the results for study identification, screening, eligibility, and final synthesis. After searching PubMed, Academic Search Premier, PsychInfo, and ERIC, 2124 studies were identified. An additional 5 articles were found after completing a hand search. After duplicates were removed, 2078 articles remained for title and abstract screening using the inclusion criteria listed above. Articles that met inclusion criteria included 263, and an additional 234 were excluded with reasons (i.e. outside age limit, study outside of the United States, and not including at least two racial/ethnic groups). A total of 29 articles were included for final synthesis.

### Study characteristics and outcomes of studies

The results of each study are shown in Tables 3 and 4. Table 3 summarizes results by study design and outcome. Across the 29 studies, 11 were cross-sectional [30, 31, 35, 40, 42, 46, 49, 52–55], 16 were cohort [22, 28, 29, 32, 33, 36–38, 41, 43–45, 47, 48, 50, 51], and 2 were experimental [34, 39]. There were no quasi-experimental study designs that met the eligibility criteria for inclusion in this review.

**Table 4:** Social Determinant of Health Categories and Outcomes

Author/Year	Social Determinants Categories			Outcomes							
	Economic	Social/Community	Neighborhood/Built Environment	Self-Efficacy	Educational Attainment	Psychological Resilience	Health Risk Behavior	Smoking	Alcohol	Substance Use	QOL^a^
Abram et al. 2017 [28]		X			X					X	
Assini-Meytin et al. 2019 [29]	X				X				X	X	
Baiden et al. 2019 [30]		X					X		X		
Bares et al. 2019 [31]			X							X	
Benner & Wang, 2014 [32]		X			X						
Benner & Wang, 2015 [33]		X							X	X	
Bohnert et al. 2017 [34]	X						X				
Brooks-Russell et al. 2019 [35]			X					X		X	
Cage et al. 2018 [36]		X			X						
Camenga et al. 2018 [37]			X					X			
Chambers et al. 2018 [38]		X					X			X	
Champion et al. 2016 [39]		X					X	X	X	X	
Chong et al. 2019 [40]		X		X							
Clark et al. 2015 [41]		X							X		
Combs et al. 2018 [42]	X				X						
Docherty et al. 2018 [43]	X						X				
Gerard & Booth 2015 [44]		X					X				
Hai 2019 [45]		X							X		
Hatchel & Marx 2018 [46]		X								X	
Hussong et al. 2018 [47]		X					X	X	X		
King 2017 [48]		X					X				
Komro et al. 2016 [49]		X							X		
Kucheva 2018 [50]			X		X						
Leventhal et al. 2018 [51]		X						X	X	X	
Lindberg et al. 2019 [52]	X						X				
Respress et al. 2018 [53]		X					X				
Santa Maria et al. 2018 [54]	X								X	X	
Wade & Peralta 2017 [55]		X							X		
Wallender et al. 2019 [22]	X										X

^a^*QOL* Quality of Life

Table 4 summarizes results by the five SDOH categories and outcomes. Across the 29 studies, 1 study examined self-efficacy [40], 6 educational attainment [28, 29, 32, 36, 42, 50], 10 health risk behavior [30, 34, 38, 39, 43, 44, 47, 48, 52, 53], 5 smoking [35, 37, 39, 47, 51] 12 alcohol use [29, 30, 33, 35, 39, 41, 45, 47, 49, 51, 54, 55], 10 substance use [28, 29, 31, 33, 35, 38, 39, 46, 51, 54], and 1 quality of life [22]. There were no studies that included psychological resilience as an outcome.

#### Social determinant of health

The Healthy People 2030 Framework categorizes SDOH into five key areas: economic stability, education access and quality, social and community context, healthcare access and quality, and neighborhood and built environment. These five key areas span a variety of topics subsequently used to identify objectives and evidence-based strategies to address public health issues [56]. The majority of the articles included for final synthesis in this review included the SDOH within the social and community context. Out of the 29 articles included for final synthesis, a total of 18 articles were categorized within the social and community context [28, 30, 32, 33, 36, 38–41, 44–49, 51, 53, 55]. Four out the 29 articles were categorized as neighborhood and built environment [31, 35, 37, 50], and 7 as economic stability [22, 29, 34, 42, 43, 52, 54]. No articles included for final synthesis included health and health care and education categories of SDOH.

## Discussion

This scoping review is one of the first to our knowledge to provide a summary of recent evidence on the role of SDOH across 9 health outcomes in US adolescence and youth aged 10–24 [12] by race and ethnicity. A reproducible search across four databases yielded 2124 articles of which 29 were included for final synthesis and extraction based on inclusion criteria.

### Summary of evidence by social determinant of health category

#### Economic stability

Economic stability refers to employment, food insecurity, housing instability, and poverty [56]. Of the 29 total studies, 7 studies examined the role of economic stability [22, 29, 34, 42, 43, 52, 54]. Outcomes examined across all studies included educational attainment, health risk behavior, alcohol and substance use, education, and quality of life. All 7 studies demonstrated racial differences in health outcomes among adolescents who were found to be economically disadvantaged. For example, economically disadvantaged African American women who participated in community-based programming, significantly decreased sedentary time and increased physical activity, compared to Hispanic women of the same age group [34].

Lindberg et al. [52] found that African American men whose mothers did not have a college degree were more likely to engage in sexual activity prior to the age of 13, compared to any other racial/ethnic and maternal education combination. For adolescents and youth who were homeless, Santa Maria et al. [54] found increased use of alcohol, marijuana, synthetic marijuana, and stimulants for those living on the street compared to those who had unstable housing or living in a shelter. These findings varied by race/ethnicity. For example, non-Hispanic white adolescents and youth had the highest lifetime use of alcohol during adolescence, synthetic marijuana, stimulants, and opioids, with significant past month use of marijuana by Hispanic and “other” race/ethnicity adolescents and youth [54]. Additionally, for adolescents and youth who were homeless and who had higher rates of adverse childhood experiences, increases were found in past use of alcohol, synthetic marijuana, and opioids, though not significant for marijuana or stimulants [54]. For adolescents and youth who experienced foster care, racial and ethnic differences were identified for rates of early pregnancy or parenthood [42]. Specifically, American Indian women and men had higher rates of early parenthood compared to those who did not identify as American Indian. Similarly, Hispanic women had significantly higher rates of early pregnancy compared to non-Hispanic women, though Hispanic men demonstrated no significant differences [42]. When considering economically disadvantaged teen fathers, Assini-Meytin et al. [29] found that African American teen fathers had lower rates of substance and alcohol use in adolescence and youth compared to non-Hispanic white and Hispanic teen fathers. By young adulthood, a greater proportion of African American and Hispanic teen fathers had not completed high school compared to non-Hispanic white teen fathers, though the difference was not significant [29].

Wallander et al. [22] found racial and ethnic differences in health-related quality of life among non-Hispanic white, African American, and Hispanic adolescents and youth, especially within early adolescence, ages 10–13. Non-Hispanic white adolescents had consistently higher quality of life, with Hispanic adolescents reporting the lowest quality of life across three grade periods, 5th, 7th, and 10th [22]. However, when adjusting for SES, differences between non-Hispanic white adolescents and African American adolescents were no longer present, though differences between non-Hispanic white and Hispanic, and African American and Hispanic remained [22]. Docherty et al. [43] examined the role of economic disadvantage on the risk of gun-carrying between African American and non-Hispanic white adolescents and did not find any racial/ethnic differences. Findings showed that peer delinquency was a stronger predictor of gun carrying at higher levels of neighborhood disadvantage, with aggression as a stronger predictor at lower levels of disadvantage [43]. African American adolescents had higher rates of neighborhood disadvantage, with a stronger predictor of peer delinquency, compared to non-Hispanic white adolescents [43].

#### Social and community context

Social and community context refers to civic participation, incarceration, discrimination, and social cohesion [56]. The majority of articles, 18, included in this review included the social and community context [28, 30, 32, 33, 36, 38–41, 44–49, 51, 53, 55]. Among these articles, 13 articles referred to social cohesion, 4 to discrimination, 1 to incarceration, and none to civic participation. The outcomes examined within the 18 articles included educational attainment, substance use, health risk behavior, alcohol use, self-efficacy, and smoking behavior. Overall, 13 of 18 articles found racial or ethnic differences in outcomes. For example, after juvenile detention, non-Hispanic white women were twice as likely to attain education compared to Hispanic or African American women [28].

Examining discrimination, adolescents and youth of color exhibited differing rates of negative health behavior related to alcohol, smoking, sexual risk behavior, and delinquent behavior when subjected to societal discrimination [51], discrimination at school [38, 53], and fear of police bias [55]. Both Leventhal et al. [51] and Respress et al. [53] found when either subjected to teacher discrimination [53] or having an increase in concern for societal discrimination [51], racial/ethnic minority adolescents and youth participated in smoking and risky sexual behavior at higher rates. Specifically, Leventhal et al. [51] found experiences of societal discrimination was associated with significantly more smoking days within the past-month for African American and Hispanic adolescents compared to other racial/ethnic groups. For students who identified as “other” race, teacher discrimination increased the likelihood for engaging in risky sexual behavior by nearly 2.2 times [53]. Chambers et al. [38] also found the more inclusive school environment, the less delinquent behavior, such as involvement in violence, was demonstrated by African American students compared to non-Hispanic white students. As the number of African American students and staff increased, and the perception of teacher discrimination decreased, the lower number of delinquent behaviors were demonstrated [38]. However, the greater amount of perceived peer inclusion, the rate of delinquent behavior increased for African American students compared to non-Hispanic white students [38]. Additionally, Wade & Peralta [55] found fear of race biased policing decreased odds of heavy episodic drinking among racial/ethnic minority adolescents. Finally, while discrimination was a risk factor for depression in Native American women compared to non-native women, it did not have a direct or indirect effect on alcohol use [49]. Overall, Komro et al. [49] found no significant differences in alcohol use for non-native and Native American women with similar predictive and protective factors, including alcohol access, parental communication, and best friend’s alcohol use.

Related to discrimination, demographic marginalization within schools was found to impact racial/ethnic differences in outcomes. Demographic marginalization refers to the proportion of students with dissimilar backgrounds [33]. For adolescents experiencing racial/ethnic marginalization within schools, ability to experience school attachment was lower, leading to more depressive symptoms, ultimately leading to higher levels of alcohol or substance use [33]. Additionally, African American students who experienced only racial/ethnic marginalization or both racial/ethnic and SES marginalization were found to have lower school attachment and educational attainment compared to all other races/ethnicities [32].

Finally, social cohesion was the most common category within the social and community context. Types, intensity, and length of time of social cohesion factors were associated with adolescent health outcomes. Parenting style and background, determined by acceptance and control, were found to contribute to racial/ethnic differences in substance use [41]. Specifically, Clark et al. [41] found no significant differences for parenting style and not engaging in heavy episodic drinking (HED) between non-Hispanic white and African American adolescents. However, for adolescents who did report HED, permissive and authoritarian parenting were risk factors for African American adolescents. Authoritarian parenting style was in turn beneficial for African American adolescents who did not report HED at age 12 [41]. Overall, higher parental socio-economic status was protective for both racial groups, with access to alcohol in the home a greater risk for African Americans [41]. Religiosity was found to be a buffering effect to alcohol and binge drinking for non-Hispanic white adolescents, compared to non-White adolescents [45].

Social interactions, both positive and negative, among peers was shown to impact health outcomes for adolescents. Chong et al. [40] found that racial/ethnic minority adolescents with greater involvement in Gay-Straight Alliances had greater race-related self-efficacy, the ability to address diversity, compared to non-Hispanic white adolescents. For both non-Hispanic white adolescents and racial/ethnic minority adolescents, having close friends who identified as racial/ethnic minorities increased self-efficacy. Furthermore, participation in discussions related to racial issues increased racial self-efficacy for non-Hispanic white adolescents, but only increased for racial/ethnic minority adolescents if discussions were frequent [40]. Gerard & Booth [44] considered the impact of individual, family, and school variables on the involvement in aggressive or delinquent behavior by non-Hispanic white and all minority adolescents. School connectedness was found to have a significant relationship with behavior for non-Hispanic white adolescents, not minority adolescents [44]. Hatchel & Marx found that school belongingness served to significantly mediate the relationship between peer victimization and drug use, also noting that while non-White adolescents experienced greater levels of victimization, there was not higher engagement in drug use [46]. In addition, Hussong et al. [47] found no racial/ethnic differences when considering social integration and depressive symptoms on substance use across varying time-points in adolescents. For adolescents who experienced bullying, physical violence, or sexual violence, differing responses of health risk behavior were found across race/ethnicity. For example, Champion et al. [39] found Mexican-American women with a history of violence were three times more likely to report substance use compared to African American women with similar histories. While Baiden et al. [30] found African American adolescents experiencing bullying or personal violence had 33% lower odds of suicidal ideation compared to non-Hispanic white adolescents when controlling for other demographic factors. However, when controlling for all predictors, these differences did not remain [30].

For adolescence and youth who have experienced abuse or neglect, racial/ethnic differences were found across outcomes. Cage et al. [36] found when considering race/ethnicity alone, there were no significant differences in educational attainment. However, when considering both race/ethnicity and gender, significant differences were found. Non-Hispanic white men and women, along with Hispanic women were over twice as likely to complete high school or obtain GED compared to African American men. Additionally, King [48] found Hispanic women had the highest rates of adolescent births compared to all other race/ethnicities, with non-Hispanic white and Asian-Pacific Islander with significantly lower birth rates. Type and occurrence of abuse, as well as time in foster care predicted the rates of early birth across all racial/ethnic groups [48]. For non-Hispanic white women, time in foster care and age of abuse were significant predictors, with reoccurrence and physical abuse significant predictors for African American and Hispanic adolescence respectively [48].

#### Neighborhood and built environment

Neighborhood and built environment refer to access to foods that support healthy eating patterns, crime and violence, environmental conditions, and quality of housing [56]. Overall, 4 articles included in this review examined racial differences in neighborhood and built environment for adolescence [31, 35, 37, 50]. Three outcomes examined within these articles included substance use, smoking, and educational attainment. Differences in outcomes among racial/ethnically diverse adolescents were mixed. Bares et al. [31] found non-Hispanic white adolescents who lived on farms had higher rates of opioid use compared to African American and Hispanic adolescents who live on farms and across all races/ethnicities living in the country or city. Considering change in past 30-day prevalence of marijuana use after retail sales became legalized, Brooks-Russell et al. [35] did not find any significant change across all racial and ethnic adolescents. Camenga et al. [37] found similar results after exposure to e-cigarette advertising, no racial differences were found in e-cigarette use.

Kucheva [50] found racial/ethnic differences when considering two different subsidized housing: public and privately managed [50]. For example, African American adolescent men in private subsidized housing and public subsidized housing were less likely to become teenage parents [50]. However, African American women were less likely to graduate high school if they lived within a privately managed subsidized housing, compared to non-Hispanic white men and women and African American men [50].

### Limitations

While this review provides a summary of recent evidence on racial/ethnic differences in SDOH and outcomes among adolescents and youth, there are several limitations that should be considered. First, the search for this review included only articles written in English, thus excluding articles that may have been relevant to understanding SDOH and adolescence published in another language. Second, studies that were disease specific or targeting a sub-population of adolescence and youth were not included, for example adolescents living with a pre-existing chronic or mental health condition. Therefore, SDOH that may contribute to specific disease occurrence or outcomes may vary from what has been presented in this summary. Finally, this review is considered narrative and cannot speak to any causal relationships.

### Implications for Public Health Education & Programming for adolescence and youth

Review of the literature demonstrates the role of multiple factors on adolescent and youth health outcomes based on the Healthy People 2030 SDOH Framework. While significant differences in outcomes were found across race/ethnicity, intersectionality of adolescent and youth identities is a critically important influence to consider for future work [57–59]. Interactions of social and structural factors, often outside the control of adolescents and youth, create a multi-dimensional understanding of adolescent and youth health behavior [57]. This review demonstrates the limited number of studies focused on SDOH domains outside of the social and community context. Identifying the impact of barriers within each domain, especially the inequitable influence across adolescent and youth populations, is crucial to addressing positive health outcomes for health during adolescence and youth. These aspects of adolescents’ and youth lives, along with structural racism [60] and disadvantage [61–63] increase the likelihood of engaging in risky health behaviors and ultimately leading to negative health outcomes during adolescence and youth and future adulthood [61, 63]. Therefore, to effectively address health and well-being, researchers, practitioners, and public health educators should consider a multidimensional and structural lens [57] when studying and developing programming for adolescent youth health [64, 65].

Public health initiatives and policies should also address social inequities to limit the accumulation of disadvantage throughout the life course [63]. School based public health initiatives in areas of sex education, safe and supportive environments and school policy improvement have been successful in addressing health inequities among adolescents [66]. Current adolescent and youth surveillance systems focus on risk behavior and school policies and practices but are limited in the inclusion of SDOH. Review of the literature demonstrates the limited data on SDOH factors, especially education and health care, relative to adolescence and youth health outcomes. SDOH indicators should be included in public health surveillance of adolescents and youth. For example, additional data related to food insecurity, housing instability, discrimination, and crime and violence could provide needed context to effectively dismantle structural barriers to positive health outcomes for adolescent and youth. Policies and programs can be tailored to specific needs of addressing adolescent and youth health equity. In addition, collaboration among the community, public health organizations, healthcare institutions and school districts is essential in addressing this multifaceted issue [67]. Finally, future research should be innovative and interdisciplinary to capture the intersectional identities of adolescence [57], health behavior, and health outcomes.

## Data Availability

Not applicable.

## References

[1] Ferrie JE, Shipley MJ, Stansfeld SA, Marmot MG (2002). Effects of chronic job insecurity and change in job security on self reported health, minor psychiatric morbidity, physiological measures, and health related behaviours in British civil servants: the Whitehall II study. J Epidemiol Community Health.

[2] Wilkinson RG, Marmot MM (2003). Social determinants of health: the solid facts.

[3] Marmot MG, Wilkinson RG (2008). Social determinants of health.

[4] Solar O, Irwin AA (2010). A conceptual framework for action on the social determinants of health: social determinants of health discussion paper 2 (policy and practice).

[5] Commission on Social Determinants of Health (2008). Closing the gap in a generation: health equity through action on the social determinants of health.

[6] Office of Disease Prevention and Health Promotion (2010). Healthy people 2020: An opportunity to address the societal determinants of health in the United States.

[7] Wilkinson RG, Marmot MG (1998). Social determinants of health: the solid facts.

[8] Braveman P, Gottlieb L (2014). The social determinants of health: it’s time to consider the causes of the causes. Public Health Rep.

[9] Norton JM, Moxey-Mims MM, Eggers PW (2016). Social determinants of racial disparities in CKD. J Am Soc Nephrol.

[10] Walker RJ, Strom Williams J, Egede LE (2016). Influence of race, ethnicity and social determinants of health on diabetes outcomes. Am J Med Sci.

[11] Office of Disease Prevention and Health Promotion. Social determinants of health. Healthy People 2030: U. S. Department of Health and Human Services. https://health.gov/healthypeople/objectives-and-data/social-determinants-health

[12] World Health Organization (2000). Health and health behavior among young people. Health Policy for Children and Adolescents, HEPCA Series 1.

[13] Blakemore SJ, Mills KL (2014). Is adolescence a sensitive period for sociocultural processing?. Annl Review of Psych.

[14] Frech A (2012). Healthy behavior trajectories between adolescence and Young adulthood. Adv Life Course Res.

[15] Viner RM, Ozer EM, Denny S (2012). Adolescence and the social determinants of health. Lancet.

[16] D’Agostino EM, Patel HH, Hansen E, Mathew MS, Messiah SE (2021). Longitudinal effects of transportation vulnerability on the association between racial/ethnic segregation and adolescence cardiovascular health. J Racial Ethn Health Disparities.

[17] Elster A, Jarosik J, VanGeest J, Fleming M (2003). Racial and ethnic disparities in health care for adolescents: a systematic review of the literature. Arch Pediatr Adolesc Med.

[18] Kim Y, Landgraf A, Colabianchi N (2020). Living in high-SES neighborhoods is protective against obesity among higher-income children but not low-income children: results from the healthy communities study. J Urban Health.

[19] Lau M, Lin H, Flores G (2012). Racial/ethnic disparities in health and health care among U.S. adolescents. Health Serv Res.

[20] Vancea M, Utzet M (2017). How unemployment and precarious employment affect the health of young people: a scoping study on social determinants. Scand J Public Health.

[21] Vartanian TP, Houser L (2020). The interactive role of SNAP participation and residential neighborhood in childhood obesity. J Children Pov.

[22] Wallander JL, Fradkin C, Elliott MN (2019). Racial/ethnic disparities in health-related quality of life and health status across pre-, early-, and mid-adolescence: a prospective cohort study. Qual Life Res.

[23] Mohajer N, Earnest J (2010). Widening the aim of health promotion to include the most disadvantaged: vulnerable adolescents and the social determinants of health. Health Educ Res.

[24] Banspach S, Zaza S, Dittus P, Michael S, Brindis CD, Thorpe P (2016). CDC grand rounds: adolescence—preparing for lifelong health and wellness. MMWR Morb Mort Wkly Report.

[25] Egerter S, Braveman P, Sadegh-Nobari T, Grossman-Kahn R, Dekker M (2011). Education and health. Exploring the social determinants of health, issue brief #5.

[26] Office of Disease Prevention and Health Promotion. Adolescent health. Healthy People 2030. U.S. Department of Health and Human Services. https://health.gov/healthypeople/objectives-and-data/browse-objectives/adolescents.

[27] Tufanaru C, Mun Z, Aromataris E, Campbell J, Hopp L, Aromataris E, Munn Z (2017). Systematic reviews of effectiveness. Joanna Briggs Institute Reviewer’s Manual.

[28] Abram KM, Azores-Gococo NM, Emanuel KM (2017). Sex and racial/ethnic differences in positive outcomes in delinquent adolescence after detention: a 12-year longitudinal study. JAMA Pediatr.

[29] Assini-Meytin LC, Garza MA, Green KM (2019). Racial and ethnic differences in teenage Fathers’ early risk factors and socioeconomic outcomes later in life. Child Adolescence Care Forum.

[30] Baiden P, Mengo C, Boateng GO, Small E (2019). Investigating the association between age at first alcohol use and suicidal ideation among high school students: evidence from the adolescence risk behavior surveillance system. J Affect Disord.

[31] Bares CB, Weaver A, Kelso MF (2019). Adolescent opioid use: examining the intersection of multiple inequalities. J of Prev Inter Comm.

[32] Benner AD, Wang Y (2014). Demographic marginalization, social integration, and adolescents’ educational success. J of Adolescence Adol.

[33] Benner AD, Wang Y (2015). Adolescent substance use: the role of demographic marginalization and socioemotional distress. Dev Psychol.

[34] Bohnert AM, Bates CR, Heard AM (2017). Improving urban minority Girls’ health via community summer programming. J Racial Ethn Health Disparities.

[35] Brooks-Russell A, Ma M, Levinson AH (2019). Adolescent marijuana use, marijuana-related perceptions, and use of other substances before and after initiation of retail marijuana sales in Colorado (2013-2015). Prev Sci.

[36] Cage J, Corley NA, Harris LA (2018). The educational attainment of maltreated adolescence involved with the child welfare system: exploring the intersection of race and gender. Chil & Adolescence Ser Rev.

[37] Camenga D, Gutierrez KM, Kong G, Cavallo D, Simon P, Krishnan-Sarin S (2018). E-cigarette advertising exposure in e-cigarette naïve adolescents and subsequent e-cigarette use: a longitudinal cohort study. Addict Behav.

[38] Chambers BD, Erausquin JT (2018). Race, sex, and discrimination in school settings: a multilevel analysis of associations with delinquency. J Sch Health.

[39] Champion JD, Young C, Rew L (2016). Substantiating the need for primary care-based sexual health promotion interventions for ethnic minority adolescent women experiencing health disparities. J Am Assoc Nurse Pract.

[40] Chong ESK, Poteat VP, Yoshikawa H, Calzo JP (2019). Fostering adolescence self-efficacy to address transgender and racial diversity issues: the role of gay-straight alliances. Sch Psychol Q.

[41] Clark TT, Yang C, McClernon FJ, Fuemmeler BF (2015). Racial differences in parenting style typologies and heavy episodic drinking trajectories. Health Psychol.

[42] Combs KM, Begun S, Rinehart DJ, Taussig H (2018). Pregnancy and childbearing among Young adults who experienced Foster Care. Child Maltreat.

[43] Docherty M, Beardslee J, Mulvey E, Schubert C, Pardini D (2018). Childhood risk factors associated with adolescent gun carrying among black and white males: an examination of self-protection, social influence, and antisocial propensity explanations. Law Hum Behav.

[44] Gerard JM, Booth MZ (2015). Family and school influences on adolescents’ adjustment: the moderating role of adolescence hopefulness and aspirations for the future. J of Adol.

[45] Hai AH (2019). Are there gender, racial, or religious denominational differences in Religiosity’s effect on alcohol use and binge drinking among adolescence in the United States? A propensity score weighting approach. Subst Use Misuse.

[46] Hatchel T, Marx R (2018). Understanding Intersectionality and Resiliency among Transgender Adolescents: Exploring Pathways among Peer Victimization, School Belonging, and Drug Use. Int J Environ Res Public Health.

[47] Hussong AM, Ennett ST, McNeish D (2018). Teen social networks and depressive symptoms-substance use associations: developmental and demographic variation. J Stud Alcohol Drugs.

[48] King B (2017). First births to maltreated adolescent girls: differences associated with spending time in Foster Care. Child Maltreat.

[49] Komro KA, Livingston MD, Garrett BA, Boyd ML (2016). Similarities in the etiology of alcohol use among native American and non-native Young women. J Stud Alcohol Drugs.

[50] Kucheva Y (2018). Subsidized housing and the transition to adulthood. Demography.

[51] Leventhal AM, Cho J, Andrabi N, Barrington-Trimis J (2018). Association of Reported Concern about Increasing Societal Discrimination with Adverse Behavioral Health Outcomes in late adolescence. JAMA Pediatr.

[52] Lindberg LD, Maddow-Zimet I, Marcell AV (2019). Prevalence of sexual initiation before age 13 years among male adolescents and Young adults in the United States. JAMA Pediatr.

[53] Respress BN, Amutah-Onukagha NN, Opara I (2018). The effects of school-based discrimination on adolescents of color sexual health outcomes: a social determinants approach. Social Work Public Health.

[54] Santa Maria DM, Narendorf SC, Cross MB (2018). Prevalence and correlates of substance use in homeless adolescence and Young adults. J Addict Nurs.

[55] Wade J, Peralta RL (2017). Perceived racial discrimination, heavy episodic drinking, and alcohol abstinence among African American and white college students. J Ethn Subst Abus.

[56] Office of Disease Prevention and Health Promotion. Social determinants of health. Healthy People 2020. U.S. Department of Health and Human Services. https://www.healthypeople.gov/2020/topics-objectives/topic/social-determinants-of-health.

[57] Lopez N, Gadsden VL (2016). Health inequities, social determinants, and intersectionality. NAM Perspectives.

[58] Evans CR (2019). Adding interactions to models of intersectional health inequalities: comparing multilevel and conventional methods. Soc Sci Med.

[59] Kern MR, Duinhof EL, Walsh SD (2020). Intersectionality and adolescent mental well-being: a Cross-nationally comparative analysis of the interplay between immigration background, socioeconomic status and gender. J Adolesc Health.

[60] Trent M, Dooley DG, Dougé J. The impact of racism on child and adolescent health. Ped. 2019;144(2). 10.1542/peds.2019-1765. 10.1542/peds.2019-1765 31358665

[61] Nurius PS, Prince DM, Rocha A (2015). Cumulative disadvantage and adolescence well-being: a multi-domain examination with life course implications. Child Adolesc Social Work J.

[62] Gustafsson PE, San Sebastian M, Janlert U, Theorell T, Westerlund H, Hammarström A (2014). Life-course accumulation of neighborhood disadvantage and allostatic load: empirical integration of three social determinants of health frameworks. Am J Public Health.

[63] Senter JP, Bucay-Harari L, Castillo-Salgado C (2020). Using social indicators to describe neighborhood-level disparities in adolescent health in Baltimore City circa 2017. J Adolesc Health.

[64] Freudenberg N, Franzosa E, Chisholm J, Libman K. New approaches for moving upstream: how state and local health departments can transform practice to reduce health inequalities. Health Educ Behav. 2015;42(1_suppl):46S–56S. 10.1177/1090198114568304. 10.1177/1090198114568304 25829117

[65] Turan JM, Elafros MA, Logie CH (2019). Challenges and opportunities in examining and addressing intersectional stigma and health. BMC Med..

[66] Centers for Disease Control and Prevention (2020). Success Stories.

[67] Center for Disease Control and Prevention. Improving School-Based Health and Education Policies. www.cdc.gov/healthyadolescence/stories/pdf/DASH-Health-Education-Policy-Success-2018.pdf

